# The first case report of complement component 7 deficiency in Qatar and a 10-year follow-up

**DOI:** 10.3389/fimmu.2023.1253301

**Published:** 2023-10-11

**Authors:** Sally Mahgoub Khalil, Sami Aqel, Dalal Sideeg Mudawi, Hassan Mobayed, Maryam Ali Al-Nesf

**Affiliations:** Allergy and Immunology Division, Department of Medicine, Hamad Medical Corporation, Doha, Qatar

**Keywords:** complement 7, C7 deficiency, complement membrane attack complex (MAC), *Neisseria meningitidis*, primary immunodeficiency diseases

## Abstract

**Introduction:**

*Neisseria meningitidis* is a significant cause of bacterial meningitis and septicemia worldwide. Recurrent *Neisseria meningitidis* is frequently associated with terminal complement protein deficiency, including Complement component 7. This report discusses the first case of C7 deficiency in Qatar.

**Case report:**

A 30-year-old Qatari man presented with a meningococcal infection, which was verified by a blood culture. He experienced two episodes of meningitis caused by an undetermined organism. His blood tests revealed low levels of CH50 and C7. His C7 gene testing revealed a homozygous mutation in exon 10 (c.1135G>C p.Gly379Arg), a mutation that has not been previously documented in Qatar. However, it has been observed in 1% of Moroccan-origin Israeli Jews who also exhibit C7 deficiency. Regular prophylactic quadrivalent vaccinations against types A, C, Y, and W-135 with azithromycin tabs were administered. Over the last 10 years of follow-up, he remained in good health, with no further meningitis episodes.

**Conclusion:**

To our knowledge, this is the first confirmed case of C7 deficiency reported in the Arabian Gulf countries. Such rare diseases should be a public health priority. Awareness among medical practitioners and the community should help with early detection of C7 deficiency and the prevention of its consequences.

## Introduction

Despite ongoing progress in understanding the pathogenesis of infection and the development of new vaccines against *Neisseria meningitidis*, it continues to be a major cause of bacterial meningitis and septicemia globally (1). *Neisseria meningitidis* is an aerobic, gram-negative diplococcus that colonizes the nasopharynx and spreads through airborne droplets. It has 13 serogroups, five of which are responsible for all human illnesses [W-135, A, B, C, and Y] (2). Once colonized, several factors aid in *Neisseria meningitidis* invasion and infection of humans, including factors related to the virulence of the organism itself, along with factors affecting host susceptibility (3). Recent genome-wide association studies have identified host factors that may contribute to disease susceptibility, and the interaction between the complement system and the meningococcus has been demonstrated to play an important role in the pathogenesis of invasive meningococcal disease (IMD) (4). The complement system comprises a strong defense against invading bacterial pathogens. It serves as the backbone of the innate immune system; it also plays a role in initiating and maintaining the acquired immune response and bridging innate and adaptive responses (5). The significance of the complement system is demonstrated by the constant correlation between a deficiency in complement components and an elevated risk of infections and/or autoimmune disorders (5, 6).

The terminal components of the complement system form the membrane attack complex (MAC), including C5b, C6, C7, C8, and C9. The MAC plays a crucial part in the host’s defensive mechanism by piercing lipid bilayers and generating transmembrane pores, which result in cell lysis and cell death (7). This innate mechanistic strategy is essential for complement-dependent bactericidal activity, especially in the body’s defense against Neisseria infection (8). The inability to form the MAC due to a deficiency in one of the terminal complement components decreases bactericidal efficacy and raises the likelihood of recurrent systemic neisserial infection. In persons with terminal complement deficits, recurrence rates for systemic neisserial infections vary from 40 to 50%, raising the possibility that each infectious attack does not provide protection against subsequent episodes of infection (9). Complement deficiencies continue to be regarded as uncommon and of limited therapeutic significance. This is a result of an inadequate level of awareness among healthcare providers, as is the case with all PIDs, as well as the fact that only a small number of facilities globally offer a thorough laboratory complement analysis. Complement deficiencies account for a range of 1–10% of primary immunodeficiency disorders (PID) (10). However, this percentage could potentially exceed 10%, as evidenced by the data from Slovenia and Israel, where it accounts for 26% and 16.2%, respectively, of all individuals with primary immunodeficiency disorders (PID) (11). Interestingly, despite the high proportion of consanguinity in the Middle East and North Africa (MENA), complement deficiencies account for less than 6% of PID in several other national registries. For instance, in Morocco, the prevalence is 3.09 per 100,000 (12), Tunisia 0.4% (13), Kuwait 4% (14), and Iran 1.4% (15). From major referral centers in Egypt, it is 1.26% (16), Saudi Arabia 5.6% (17), and Oman 3.5% (18). There has not been a previously reported case of complement insufficiency in Qatar (19).

This paper details the first inherited C7-deficient case in Qatar, with a history of recurrent meningitis and follow-up over a 10-year period.

## Case description

A 30-year-old Qatari male patient presented to the emergency department in May 2012 at the age of 20, complaining of a one-day fever, sore throat, and headache. The fever was associated with a frontal headache, neck pain, and mild confusion, for which meningitis was suspected. There were no respiratory, gastrointestinal, or urinary symptoms. Regarding his past history, he had had two previous non-complicated bacterial meningitis at the ages of 7—but there were no available details in his records about that incident—and 18, for which he was empirically treated to cover meningococcal infection, as no organism’s growth was identified in the blood or cerebrospinal fluid. He made a complete recovery. Family history revealed the death of a younger sibling at the age of 15 in 2009 due to meningitis, and no workup for him was done at that time. The patient is part of a sibling group consisting of two older sisters, the patient himself, a deceased brother, and a younger brother. There is no documented medical history of meningitis or any chronic disease among any of his currently living siblings. The patient was admitted to the Medical Intensive Care Unit. On admission, the patient’s temperature was 36.5°C, his heart rate was 100 beats per minute, his respiratory rate was 24 breaths per minute, his blood pressure was 112/72 mmHg, and his oxygen saturation was 94%. Physical examination revealed neck rigidity along with positive meningeal irritation signs (neck stiffness & Brudzinski sign) (20). Examination of the chest, heart, abdomen, and musculoskeletal system was normal.

The blood tests showed elevated white blood cells (40.8 X10^3^/ul (4.0–10.0), neutrophils 37.4 X10^3^/ul/92.8% (7.4-10.4 X10^3^/ul); lactic acid 5.88 mmol/l (arterial 0.5-1.6-venous 0.5-5.5), procalcitonin 37.55 ng/ml (<0.5ng/ml related to low risk of severe sepsis, >2 related to high risk of severe sepsis), and a C reactive protein (CRP) of 88 mg/l (0<5). *Neisseria meningitidis* was found in a blood culture but not in a CSF culture. The bacteria (undetermined serotypes) were sensitive to ciprofloxacin, ceftriaxone, penicillin, and rifampin.

The complement system function assessment with total hemolytic complement (CH50) by hemolytic assay was low 7 U/ml (30-75 U/ml). The Alternative Complement Pathway, Functional Serum AH50, was low as well <10% of normal (normal is >50%). The C3 and C4 levels were normal. Further analysis of individual terminal pathway components (performed in the Mayo clinic reference lab) C5, C6, C7, C8, and C9 was carried out using an antigen assay that quantitates the amount of protein and was confirmed by the Automated Liposome Lysis Assay. There was a complete absence of the C7 component, which was undetectable at less than 5 U/mL (normal, 36–60 U/mL), and normal levels of the other components, including C6. The concurrent C6-C7 deficiency that has been previously observed (21) is excluded by the patient’s normal C6 levels. Immunoglobulin levels were normal, apart from IgG2 at 98.6 mg/dL (169–786). No other immunological abnormalities were found, including antinuclear antibodies (ANA), antineutrophil cytoplasmic antibodies (ANCA), extractable nuclear antigen antibodies (ENA), antibacterial antibodies, and lymphocyte subsets.

As a case of recurrent meningitis, he was treated with intravenous Meropenem (2 gm/8 hrs) for two weeks and dexamethasone 0.15 mg/kg for six hours a day for seven days. He demonstrated a successful recovery with no complications from the infection or medication. Upon discharge, he was given ciprofloxacin 500 mg orally for one day. All of his close contacts were given a prophylactic dose of ciprofloxacin 500 mg as a single dose.

A peripheral blood sample of the patient was sent for a genetic study, C7 gene analysis was performed by Centogene AG, and the C7 gene was analyzed by PCR and sequencing of both DNA strands of the entire coding region and the highly conserved exon-intron splice junction. The reference sequence of the C7 gene is NM_000587.2. The result showed a homozygous mutation in exon 10 of the C7 gene (c.1135G>C p.Gly379Arg) that is inherited in an autosomal recessive manner (**Table 1**). Fernie (1996) described this mutation as a disease-causing cause of C7 deficiency (11).

**Table 1:** Terminal complement component concentrations with other immunological assays done for the patient with C7 deficiency.

Complement Component	Concentration	Normal value	Unit value	Assay Type
CH50	7 U/ml	30-75	U/ml	Haemolytic Assay
AH50	<10% of normal	>50% of normal		
C3	112	90-180	mg/dl	Nephelometry
C4	24.4	10-40		
C5	46	29-53	U/ml	By antigen assays that quantitate the amount of the protein and confirmed by the Automated Liposome Lysis Assay (Mayo clinic test).
C6	48	32-57		
**C7**	**<5**	36-60		
C8	50	33-58		
C9	52	37-61		
Immunoglobulin level				
IgG	1110	700-1600	mg/dl	Nephelometry
IgA	74.9	70-400		
IgM	54.6	40-230		
IgG1	838	400-1011		
**IgG2**	**98**	169-786		
IgG3	52	11-85		
IgG4	<6.0	3-201		
IgE	72.8	0-114	Ku/l	ImmunoCap
Anti-bacterial antibodies				
Anti- pneumococcus IgG	39.13	3.3-270		ELISA Assay
Anti-haemophilus IgG	>9	0.11-9		
Anti-Tetanus Toxoid IgG	3.76	0.01-1.5		
Other immunological tests				
ANA	Negative	1:40		IFA
Genetic Testing				
WES	Homozygous mutation in exon 10 of the C7 gene (c.1135G>Cp.Gly379Arg)			

As a preventative measure, the patient was planned to receive the meningococcal vaccine every three years, along with azithromycin three times per week. He received the meningococcal polysaccharide vaccine (MPSV-4), followed by the quadrivalent meningococcal vaccine (protecting against 4 types of meningococcal bacteria: types A, C, Y, and W-135) in 2012 and again in 2015 and 2019. He joined the military in 2012 and experienced no further infections. The plan was to screen all family members with CH50 and genetic tests. Unfortunately, all the family members were unwilling to undergo any screening tests.

## Discussion

The identification of a complement deficiency in patients with invasive meningococcal disease (IMD) is important since it may lead to the avoidance of future episodes. Up to 20% of IMD patients lack any of the terminal complement components or properdin (22). Interestingly, complement deficiency individuals have a median age of 17 when they experience their first episode of meningococcal disease, whereas those with adequate complement levels experience their first episode at a median age of 3 (23). This could be explained by the fact that while the risk of meningococcal infection decreases with age as serum bactericidal activity matures, meningococcal infections are a lifetime risk for those with late complement component deficiencies (LCCD) (24). In the case we are presenting, the patient developed meningococcal meningitis for the first time at the age of 20. Other notable differences between complement-sufficient and complement-deficient individuals include lower mortality rates as well as a milder course of disease in complement-deficient individuals, despite the high recurrence rate associated with late complement component-deficient (LCCD) individuals (10). Some researchers think that the lower death rate for LCCD patients is due to “ascertainment bias,” which is when complement testing is done on people who survived meningococcal disease but not on people who died from the disease (24). The likely cause for the slower disease progression in LCCD patients is that individuals with LCCD retain the bacterial cell membrane in the absence of MAC, resulting in decreased endotoxin leakage, the leading cause of septic shock, cerebral edema, coagulation issues, and death (25). Furthermore, meningococcal infection episodes occur in LCCD patients in the presence of protective antibodies, allowing for opsonization and phagocytosis of a portion of the organism (24). This could explain our patient’s three uncomplicated meningitis attacks and good response to antibiotics. Regarding the isolated meningococci serotypes, earlier studies found no changes in the serotypes or subtypes of meningococci between complement-deficient and complement-sufficient patients with the condition and noticed that infections induced by new strains accounted for the great majority of recurrences (26). Recent research, on the other hand, indicates that terminal complement deficiencies increase the risk of invasive meningococcal disease (IMD) caused by uncommon or minor meningococcal serogroups, such as Y and W-135. However, the capsular group or clonal complex has not been linked to specific terminal complement deficiencies (1, 22, 27). Complement deficiency can be screened by measuring the functional activity of the classical pathway (CH50), the alternative pathway (AH50), and the lectin pathway (LP) (28). Both CH50 and AH50 are reduced in the presence of a complement protein or regulatory protein deficiency that affects the terminal pathway (29). The patient’s CH50 and AH50 levels in this case report were low, which is consistent with a deficit in the terminal pathway. Therefore, a late terminal complement deficiency was suspected based on a positive family history of a similar condition, as well as normal C3 and C4 levels, which ruled out alternative pathway diseases. As a consequence, the levels of each late complement component (C5–C9) were quantified, demonstrating the absence of the C7 component. The diagnosis was then confirmed by C7 gene testing, which indicated a pathogenic mutation.

C7 deficiency is a genetically defined complement component that is inherited in an autosomal recessive pattern (24). It is reported to have a high prevalence (1.1%) in the Israeli Moroccan Jewish population (30). The C7 gene, which is composed of 18 exons, maps to 5p12–14, the same position as the C6 and C9 genes. C7’s single polypeptide chain contains 821 amino acid residues and is biochemically and physically related to other late complement components, particularly C6 (31).

More than 22 different molecular defects leading to total or subtotal C7 deficiency defects have been reported in the literature (31). To the best of our knowledge, two pathogenic variants have so far been found in MENA regions. The 1135G>C p.Gly379Arg in Exon 10 mutation, which was found in our reported case, causes C7 deficiency in 1% of the Israeli Jewish population of Moroccan origin (32). A second pathogenic mutation, 1135G>C p.Gly357R in exon 9 of the C7 gene, was also found in Israel in a highly inbred Arab population (33). This is the first report of C7 deficiency in Qatar and other Gulf countries related to this mutation.

Education on the increased risk of severe, life-threatening infections and the significance of seeking medical assistance early for febrile disorders is essential for people with complement deficiency. Moreover, preventive measures, such as vaccination, which can be used with or without antibiotics, are crucial (10). Vaccination (tetravalent meningococcal vaccine A/C/W/Y and the recently available vaccine against serogroup B) is recommended, even in asymptomatic persons (22). The decision to provide vaccinated patients with additional antibiotic prophylaxis (penicillin or macrolide-based) depends on the patient’s risk profile. Adding antibiotic prophylaxis may be an option for patients who have a history of recurrent infections and are susceptible to microorganisms (for instance, individuals who live in endemic areas or work in high-risk professions, such as nursery care) (10). Given the patient’s history of recurrent meningitis in this reported case, the decision was made to initiate both vaccination (quadrivalent meningococcal vaccine against types A, C, Y, and W-135) and antibiotics (azithromycin three times a week). The patient was advised that, despite the fact that vaccination did not completely protect against meningococcal disease, the vaccinated group had a considerably lower prevalence of the condition than the unvaccinated group (22). Since he started taking both medicines after his last episode of meningitis ten years ago, he has been healthy and hasn’t had any more infections.

## Conclusion

Among the uncommon complement deficiencies, C7 deficiency is one of the rarest and can be missed easily if not considered in the differential diagnosis by the treating physician. We recommend screening using the CH50 test for all patients following the first attack of meningitis at any age beyond the window of susceptibility (i.e., above three years), especially in areas with low prevalence and for any individual of any age who has a family history of LCCD or meningococcal infections. Moreover, the patient’s family should be counseled and screened if the inherited deficiency is confirmed. Education and counseling to seek immediate medical help for febrile disease in families known to have terminal complement deficiencies should be emphasized during routine clinic visits. Finally, the patient with a C7 deficiency should be counseled to receive quadrivalent meningococcal vaccines every 3–5 years as a minimal preventive measure to augment the immune system and reduce the risk of infection.

## Data availability statement

The original contributions presented in the study are included in the article/supplementary material. Further inquiries can be directed to the corresponding author.

## Ethics statement

Written informed consent was obtained from the individual for the publication of any potentially identifiable images or data included in this article because consent was obtained from the patient but not uploaded with the case report.

## Author contributions

SK: Conceptualization, Data curation, Formal Analysis, Investigation, Methodology, Resources, Software, Supervision, Writing – original draft, Writing – review & editing. DM: Data curation, Investigation, Writing – review & editing. SA: Data curation, Investigation, Methodology, Writing – review & editing. HM: Formal Analysis, Supervision, Writing – review & editing. MA: Data curation, Formal Analysis, Supervision, Writing – review & editing.
